# Supplementary material for: Alternative Hapten Design for Zearalenone Immunoreagent Generation
Source: Toxins (Basel). 2022 Mar 2;14(3):185. doi: 10.3390/toxins14030185 (PMC8953469; doi:10.3390/toxins14030185)
Supplement: Supplementary file 1 [file toxins-14-00185-s001.zip › toxins-1605169-supplementary.pdf]

Supplementary Materials: Alternative Hapten Design for Zearalenone Immunoreagent Generation

Antonio Abad-Fuentes, Consuelo Agulló, Daniel López-Puertollano, Ismael Navarro-Fuertes, Antonio Abad-Somovillaand and Josep Vicent Mercader

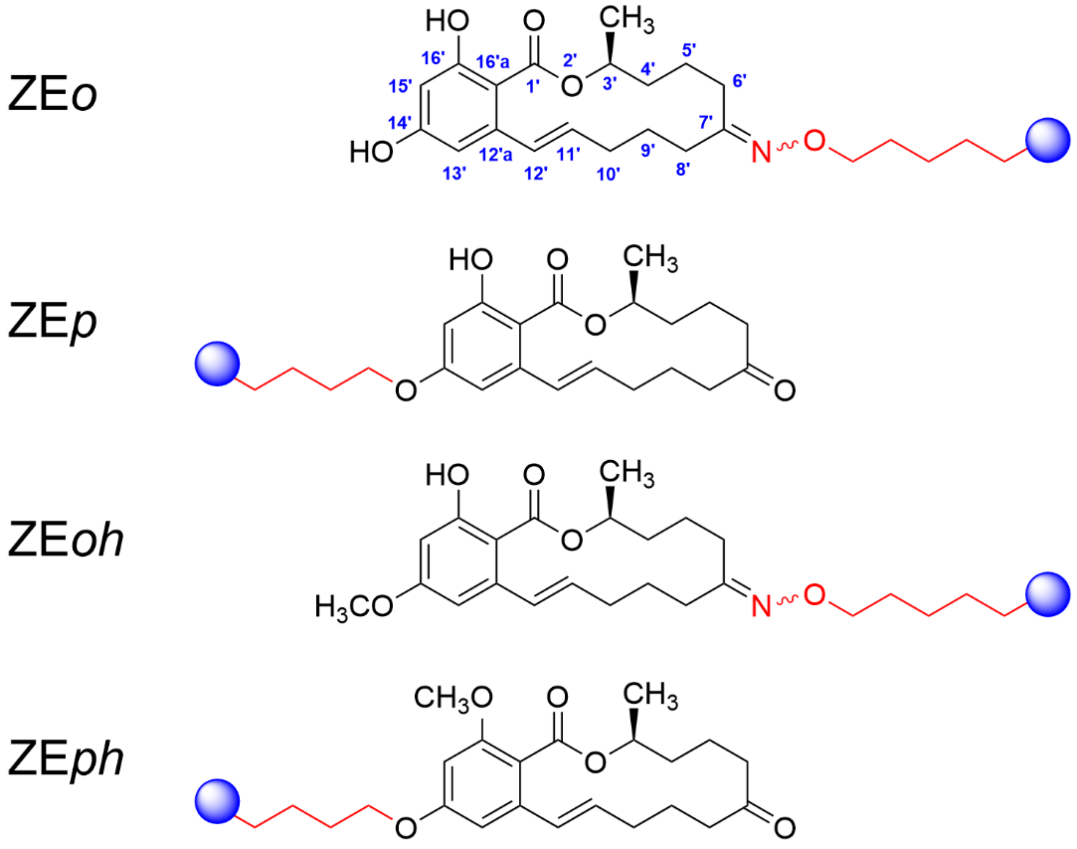

Figure S1. Hapten chemical structures in the prepared bioconjugates.

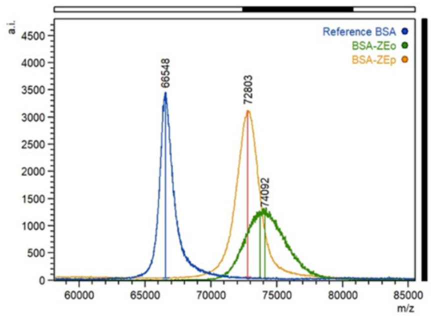

	m/z	Δm/hapten	Δ(*m/z*)	MR	MR_o
**BSA**	66548	–	–	–	–
**BSA-ZE*o***	74092	429	7544	17.6	30
**BSA-ZE*p***	72803	400	6225	15.6	30

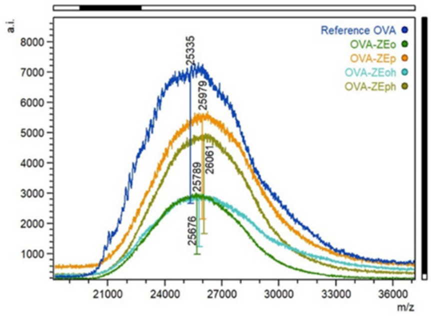

	m/z	Δm/hapten	Δ(*m/z*)	MR	MR_o
**OVA**	25335	–	–	–	–
**OVA-ZE*o***	25676	429	341	1.6	10
**OVA-ZE*p***	25979	400	644	3.2	10
**OVA-ZE*oh***	25789	443	454	2.0	10
**OVA-ZE*ph***	26061	414	726	3.5	10

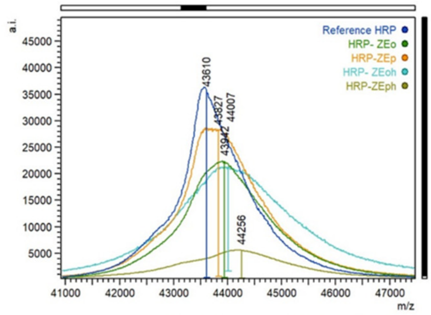

	m/z	Δm/hapten	Δ(*m/z*)	MR	MR_o
**HRP**	43610	–	–	–	–
**HRP-ZE*o***	43942	429	332	0.8	8
**HRP-ZE*p***	43827	400	217	0.5	8
**HRP-ZE*oh***	44007	443	397	0.9	12
**HRP-ZE*ph***	44256	414	646	1.6	12

Figure S2. MALDI-TOF/TOF mass spectra (singly charged ions for BSA and HRP conjugates and doubly charged ions for OVA conjugates) of BSA, OVA, and HRP (blue) and the corresponding conjugates with haptens ZE*o* (green), ZE*p* (orange), ZE*oh* (cyan), and ZE*ph* (olive green). MR_o and MR: initial and final hapten-to-protein molar ratio, respectively.

Table S1. Antibody characterization by competitive ELISA using the bioconjugates of haptens ZE*o* and ZE*p* as heterologous conjugates (n = 3).[a]

Ab	d-cELISA			i-cELISA		
	**[Ab]**[b]	**[HRP]**[c]	IC_{50}[d]	[Ab]	[OVA]	IC_{50}
ZE*o*#1	-[e]	-	-	-	-	-
ZE*o*#2	-	-	-	-	-	-
ZE*p*#1	-	-	-	45	1000	1.1
ZE*p*#2	-	-	-	5	1000	2.9

[a] A_{max} values were between 0.5 and 1.5. [b] Antibody dilution factor $\times 10^{-3}$. [c] Bioconjugate concentrations are in ng/mL. [d] Values are in nM units. [e] No signal was observed.

Table S2. Cross-reactivity (%) using ZE*o*-derived antibodies (n=2).

**OVA conj.**	**Antibody**											
	**ZE*o*#1**						**ZE*o*#2**					
	**ZEN**	**ZAN**	**α-ZEL**	**β-ZEL**	**α-ZAL**	**β-ZAL**	**ZEN**	**ZAN**	**α-ZEL**	**β-ZEL**	**α-ZAL**	**β-ZAL**
ZE*o*	100	25.9	30.1	9.9	12.0	3.9	100	50.3	68.0	26.6	42.0	9.3
ZE*oh*	100	35.1	57.9	15.4	12.1	5.3	100	12.2	93.7	18.1	9.7	5.2
ZE*p*	100	-	-	-	-	-	100	-	-	-	-	-
ZE*ph*	100	-	-	-	-	-	100	-	-	-	-	-

Table S3. Cross-reactivity (%) using ZE*p*-derived antibodies (n=2).

**OVA conj.**	**Antibody**											
	**ZE*p*#1**						**ZE*p*#2**					
	**ZEN**	**ZAN**	**α-ZEL**	**β-ZEL**	**α-ZAL**	**β-ZAL**	**ZEN**	**ZAN**	**α-ZEL**	**β-ZEL**	**α-ZAL**	**β-ZAL**
ZE*o*	100	32.8	53.9	1.7	12.4	0.6	100	48.6	4.5	0.5	1.0	0.1
ZE*oh*	100	53.6	43.1	2.1	9.6	0.8	100	38.1	5.8	0.4	1.5	0.3
ZE*p*	100	33.5	1.9	0.4	0.6	0.1	100	52.5	4.0	0.6	1.3	0.3
ZE*ph*	100	53.8	0.8	0.4	0.2	0.2	100	42.1	3.3	1.0	1.0	0.2

Copies of ^{1}H and ^{13}C NMR spectra of haptens.
